# Multifaceted Genome Control by Set1 Dependent and Independent of H3K4 Methylation and the Set1C/COMPASS Complex

**DOI:** 10.1371/journal.pgen.1004740

**Published:** 2014-10-30

**Authors:** Irina V. Mikheyeva, Patrick J. R. Grady, Fiona B. Tamburini, David R. Lorenz, Hugh P. Cam

**Affiliations:** 1 Biology Department, Boston College, Chestnut Hill, Massachusetts, United States of America; University of California San Francisco, United States of America

## Abstract

Histone modifiers are critical regulators of chromatin-based processes in eukaryotes. The histone methyltransferase Set1, a component of the Set1C/COMPASS complex, catalyzes the methylation at lysine 4 of histone H3 (H3K4me), a hallmark of euchromatin. Here, we show that the fission yeast *Schizosaccharomyces pombe* Set1 utilizes distinct domain modules to regulate disparate classes of repetitive elements associated with euchromatin and heterochromatin via H3K4me-dependent and -independent pathways. Set1 employs its RNA-binding RRM2 and catalytic SET domains to repress *Tf2* retrotransposons and pericentromeric repeats while relying on its H3K4me function to maintain transcriptional repression at the silent mating type (*mat*) locus and subtelomeric regions. These repressive functions of Set1 correlate with the requirement of Set1C components to maintain repression at the *mat* locus and subtelomeres while dispensing Set1C in repressing *Tf2s* and pericentromeric repeats. We show that the contributions of several Set1C subunits to the states of H3K4me diverge considerably from those of *Saccharomyces cerevisiae* orthologs. Moreover, unlike *S. cerevisiae*, the regulation of Set1 protein level is not coupled to the status of H3K4me or histone H2B ubiquitination by the HULC complex. Intriguingly, we uncover a genome organization role for Set1C and H3K4me in mediating the clustering of *Tf2s* into *Tf* bodies by antagonizing the acetyltransferase Mst1-mediated H3K4 acetylation. Our study provides unexpected insights into the regulatory intricacies of a highly conserved chromatin-modifying complex with diverse roles in genome control.

## Author Summary

Methylation of histone H3 at lysine 4 (H3K4me) is a well-documented mark associated with euchromatin. In this study, we investigate the contributions of the histone methyltransferase Set1 (KMT2) and its associated Set1C/COMPASS complex in the fission yeast *Schizosaccharomyces pombe* to histone H3 lysine 4 methylation (H3K4me), transcriptional repression, and genome organization. We show that Set1 exhibits multiple modes of transcriptional repression at different types of repetitive elements, requiring distinct domains of Set1 and other Set1C subunits. Despite high conservation of subunits between the *S. pombe* and *S. cerevisiae* Set1C complexes, there are considerable differences in contributions to H3K4me by several individual subunits. Furthermore, unlike a recent report in *S. cerevisiae*, the abundance of Set1 proteins in *S. pombe* is generally not coupled to either the status of H3K4 methylation or H2B ubiquitination, further highlighting critical differences in Set1 regulation between the two yeast species. We describe a role for the Set1C complex in the nuclear organization of dispersed retrotransposons into *Tf* bodies. Set1C maintains *Tf* body integrity by employing H3K4me to antagonize the activities of the H3K4 acetyltransferase Mst1. Collectively, our findings dramatically expand the regulatory landscape controlled by the Set1C complex, an important and highly conserved chromatin-modifying complex with diverse roles in genome control and development.

## Introduction

In eukaryotic cells, DNA-based processes operate within the context of a chromatin template [1], [2]. Chromatin-modifying complexes targeting select residues of histones for posttranslational modifications exert various levels of genome control including chromatin assembly, transcription, DNA repair, replication and recombination [1], [3]. Furthermore, modified histone marks contribute to shaping the genome landscape into distinct chromatin domains. Most notable is the methylation of histone H3 at lysine 4 (H3K4me), which distinguishes euchromatin from heterochromatin, which is marked by H3 lysine 9 methylation (H3K9me) [4], [5]. H3K4me can exist as mono- (H3Kme1), di- (H3K4me2), or tri- (H3K4me3) methylation, and is catalyzed by a number of SET-containing histone methyltransferases that are parts of Set1C/COMPASS and MLL complexes [6], [7]. The roles of individual Set1C/COMPASS subunits have been revealed through studies primarily in the budding yeast *Saccharomyces cerevisiae*, with loss of individual subunits of Set1C having different effects on the stability of the complex and the states of H3K4me [8], [9], [10], [11]. Interestingly, there is a positive codependency between the levels of H3K4me and those of Set1 proteins [12]. Consistent with the prevalent enrichment of H3K4me2 and H3K4me3 throughout the gene-rich euchromatin [13], [14] Set1C has been shown to localize to active RNA Polymerase II (Pol II) genes [15], [16]. However, accumulating evidence implicates a variety of genetic elements under the repressive control of Set1 and H3K4me. In budding yeast the silencing of *Ty1* retrotransposons [17], long noncoding RNAs [18], and antisense regulatory noncoding RNAs [19] requires Set1 and H3K4me. In addition, transcriptional profiling analysis of Set1C/COMPASS mutants supports repressive roles for H3K4me3 at ribosomal genes during multiple stresses [20] and for H3K4me2 and H3K4me3 through promotion of 3'end antisense transcription [21].

In the fission yeast *Schizosaccharomyces pombe*, Set1 (KMT2) is the sole histone methyltransferase responsible for H3K4me [22], [23]. Biochemical purification identified Set1 as the core subunit of the Set1C complex whose components have orthologs in budding yeast and humans [24]. However, the contributions of Set1 and individual Set1C subunits to H3K4me and their roles in transcriptional repression are not well-characterized in *S. pombe*. Previous genome-wide mapping shows that the *S. pombe* genome is dominated by a euchromatin landscape marked with H3K4me2 [14]. Heterochromatin domains distinguished by H3K9me are restricted to prominent genome landmarks including pericentromeres, subtelomeres, ribosomal DNA arrays, and the silent mating-type locus (*mat*) [14]. These domains contain repetitive elements that help direct RNAi-mediated heterochromatin assembly [25]. The *S. pombe* genome also contains repetitive elements in the forms of long terminal repeat (LTR) *Tf2* retrotransposons and their LTR remnants interspersed across euchromatin and not normally targeted for heterochromatic silencing [14], [26]. Instead, repression of *Tf2* retrotransposons, which are enriched for H3K4me2 [14], requires Set1 [27].

In this study, we investigate the contributions of various protein domains of Set1 and its associated Set1C subunits to H3K4me and their roles in the regulation of repetitive elements associated with euchromatin and heterochromatin. We find that *S. pombe* Set1 possesses multiple modes of regulation that are dependent and independent of H3K4me and Set1C. Set1-mediated repression of *Tf2s* and pericentromeric repeats is maintained in mutant cells deficient in Set1C subunits or Set1 domain mutants with defects in H3K4me activity. In contrast, intact H3K4me by the Set1C complex is required to maintain repression at the *mat* and subtelomeric regions. We show that the contributions of several individual Set1C subunits to the levels of H3K4me and Set1 proteins are notably different from those of *S. cerevisiae* orthologs. Whereas a recent study identifies a feedback mechanism between H3K4me and Set1 protein levels in *S. cerevisiae*, we find that the stability of Set1 proteins is not coupled to the levels of H3K4me or HULC complex-mediated H2B ubiquitination. Finally, we describe a surprising role for the Set1C complex in the nuclear organization of *Tf2* elements into *Tf* bodies. Set1C employs H3K4me to limit the levels of H3K4 acetylation at *Tf2s* by antagonizing the function of the histone H3K4 acetyltransferase Mst1. Our study considerably expands the regulatory repertoire of an important histone modifier and highlights the multifaceted function by a highly conserved chromatin-modifying complex with diverse roles in genome control.

## Results

### Set1 domains and methyltransferase activity play distinct roles in transcriptional silencing of *Tf2* retrotransposons and heterochromatic repeats

**Figure 1:**
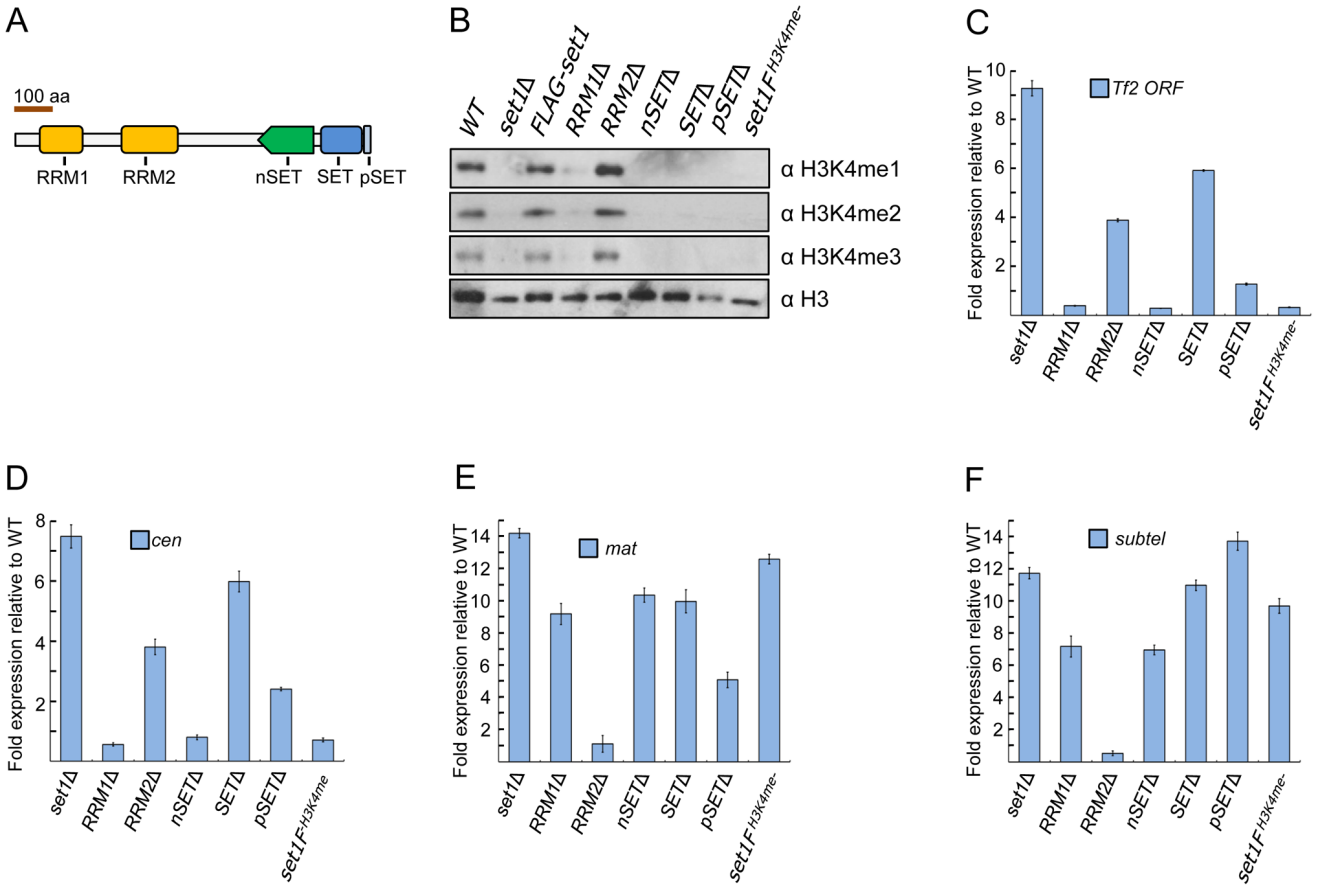
Set1 represses different classes of repetitive elements via distinct functional domains. (A) Schematic of *S. pombe* Set1 protein domain architecture. (B) H3K4 methylation (H3K4me) in *set1* domain mutants. Mono (H3K4me1), di (H3K4me2), and tri (H3K4me3) methylation of H3K4 was analyzed from histone extracts of indicated *set1* mutant strains by western blotting. Full-length FLAG-set1 and the indicated deleted domain mutants of *set1* contain an N-terminal FLAG (3×) epitope. *set1F^H3K4me-^* denotes an H3K4me null mutant due to the presence of a FLAG (3×) epitope at the C-terminus of *set1*. (C) *Tf2* repression requires intact RRM2 and SET domains. (D–F) Set1 represses heterochromatic loci dependent and independent of H3K4me. Expression of *Tf2* ORF and heterochromatic loci was analyzed by qRT-PCR. Fold changes relative to wildtype were normalized by *act1* expression. (s.d., error bars; *n* = 3). Pericentromeric repeat *dg* (*cen*), silent mating type *cenH* (*mat*), subtelomeric *prl70* (*subtel*).

The protein architecture of fission yeast Set1 is highly conserved [22], [24], containing two putative RNA-recognition motifs (RRMs) termed RRM1 and RRM2 near the N-terminus [28], [29], an nSET domain responsible for interaction with certain COMPASS subunits [11], [30], a catalytic SET domain and a short post-SET (pSET) domain near its C-terminus [28] (Figure 1A). Previous studies from budding yeast have shown that H3K4me is affected by the loss of various domains of Set1 [11], [28], [29], [31]. We examined the status of H3K4me in *S. pombe* mutant strains lacking individual domains of *set1*. Loss of RRM1 abolished H3K4me3, substantially diminished H3K4me2 [22], and slightly decreased H3K4me1 compared to wildtype (Figures 1B and S1). An RRM2 deletion resulted in no appreciable decrease in H3K4me levels. Cells expressing Set1 with a deleted nSET, SET or pSET domain displayed a complete loss of H3K4me. *S. cerevisiae* cells expressing Set1 with a C-terminal TAP epitope showed reduced H3K4me levels [32] while an affinity-purified *S. pombe* equivalent Set1-TAP protein retained *in vitro* H3K4me activity [24]. We found that H3K4me was completely abolished in cells containing a FLAG (3×) epitope attached to the C-terminus of Set1 (*set1F^H3K4me-^*), likely a result of the epitope interfering with the interaction of the SET or pSET domain with the H3K4 substrate [33], [34].

Deletion of a protein domain could affect the stability of Set1 proteins [11], [12]. To examine this possibility, we constructed strains expressing either full-length or domain-deleted Set1 that contains a fused FLAG epitope at the N-terminus of Set1. Unlike certain *S. cerevisiae* domain mutants in which Set1 protein level was undetectable [12], our western blot analysis readily detected Set1 expression of all domain mutants (Figure S2). However, there were noticeably reduced levels of Set1 proteins lacking either the RRM1 or nSET domain, suggesting that H3K4me defects observed in *RRM1* and *nSET* mutants could partly be due to reduced amount of Set1 proteins in these mutants. Slight decreases in Set1 protein levels were seen in mutants deficient in *RRM2*, *SET*, and *pSET* domains (Figure S2).

We next performed reverse transcription followed by realtime PCR (qRT-PCR) analysis to examine the effect of various *set1* mutations on *Tf2* expression. Cells deficient in H3K4me (*set1F^H3K4me-^*) or lacking either the RRM1, nSET or pSET domain exhibited little change in transcript levels of *Tf2s* (Figure 1C). However, *Tf2* expression was substantially increased in cells lacking either the RRM2 or SET domain. These results support a catalytic mode of Set1-mediated repression independent of H3K4me that requires intact RRM2 and SET domains. Loss of *set1* has been shown to compromise centromeric and telomeric silencing of a reporter gene [23]. We performed qRT-PCR in *set1* mutant strains to assess the status of transcription at known heterochromatic regions. Intriguingly, similar to *Tf2s*, derepression of pericentromeric repeats was observed only in *set1*Δ and in cells lacking either the RRM2 or SET domain (Figure 1D), suggesting a common mode of Set1-mediated repression for retrotransposons and pericentromeric repeats. In contrast, derepression at the *mat* locus and subtelomeres was observed in all mutants with defects in H3K4me including *set1F^H3K4me-^* (Figures 1E and 1F).

The *S. pombe* genome encodes three functional copies of histone H3 at distinct loci [35], [36]. We previously observed that repression of *Tf2s* was maintained in H3K4 mutants in which lysine 4 on all three copies of histone H3 was substituted for either alanine (H3K4A) or arginine (H3K4R) [27]. A previous study reported upregulation of pericentromeric repeats in a H3K4R mutant containing only one functional copy of histone H3 [37]. However, we saw little change in repression at the three major heterochromatin domains in our H3K4 mutants (Figure S3), suggesting a role for histone gene dosage acting together with modifications at certain histone residues (i.e., H3K4) to maintain heterochromatic silencing. Collectively, these results suggest that Set1 utilizes distinct modes to repress different classes of repetitive elements via H3K4me -dependent and -independent pathways.

### Loss of the RRM1 or nSET domain impairs Set1 localization at a house-keeping gene and *Tf2s*, but is dispensable at pericentromeric repeats

**Figure 2:**
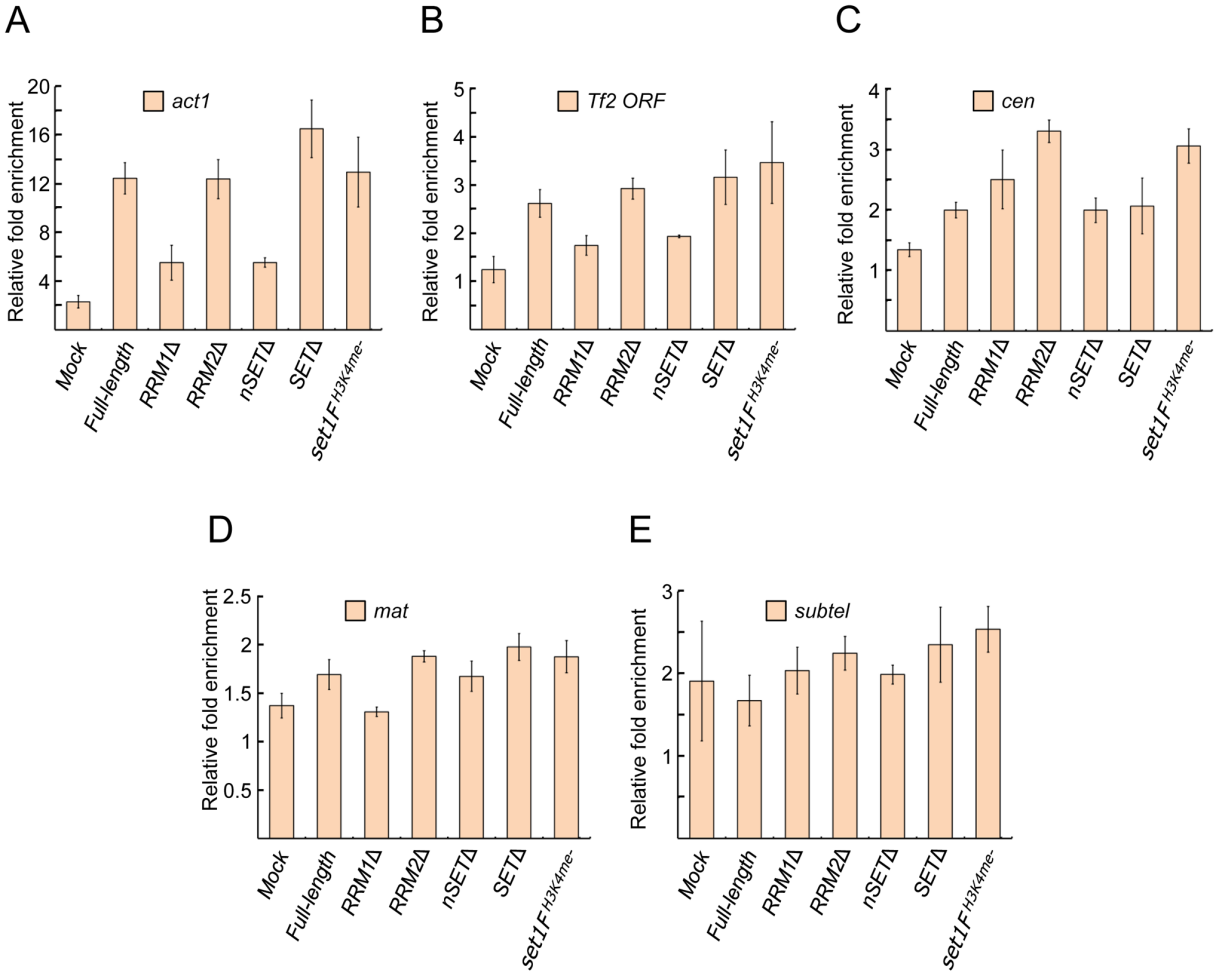
Set1 localization at euchromatic and heterochromatic targets is largely unaffected by certain domain deletions or H3K4me status. Enrichment of Set1 was determined by chromatin immunoprecipitation (ChIP) followed by quantitative PCR (qPCR) using primers targeting (A) the highly expressed actin gene (*act1*) promoter, (B) the 5′ end of *Tf2* open reading frames, (C) the pericentromeric *dg* repeat of chromosome II (*cen*), (D) the silent mating type *cenH* (*mat*), and (E) the chromosome I subtelomeric *prl70* (*subtel*). Mock denotes untagged wildtype Set1 strains. Percent enrichment of target amplification compared to input (whole cell extract) was calculated using the 2^−ΔΔCt^ method following normalization by primers targeting mitochondrial DNA (see Materials and Methods). (s.d., error bars; n = 2 biological×2 qPCR replicates).

We have previously shown that Set1 localizes at *Tf2s* [27]. Whether Set1 localizes at heterochromatic repeats is not known. We utilized chromatin immunoprecipitation (ChIP) to monitor Set1 enrichment at known euchromatin and heterochromatin targets in *set1* mutant strains. In strains deficient in either the RRM1 or nSET domain, there was reduced Set1 enrichment at the housekeeping actin gene *act1* and *Tf2s* (Figures 2A and 2B). Loss of the RRM2, SET domain or H3K4me function did not appear to hamper Set1 localization at these elements. Surprisingly, we detected Set1 localization at pericentromeric repeats (Figure 2C). Unlike the two examined euchromatic targets (i.e., *act1* and *Tf2s*), Set1 localization at pericentromeric repeats was generally not affected in strains deficient in any one of the domains. We did not detect Set1 enrichment at the *mat* locus or subtelomeric repeats either in wildtype or domain mutants (Figures 2D and 2E). These results suggest that the absence of H3K4me does not adversely affect Set1 localization at certain euchromatic and heterochromatic targets and that H3K4me-dependent repression might not require stable interaction of Set1 with its target loci.

### *S. pombe* Set1C/COMPASS subunits affect H3K4 methylation distinctly from their *S. cerevisiae* orthologs

**Figure 3:**
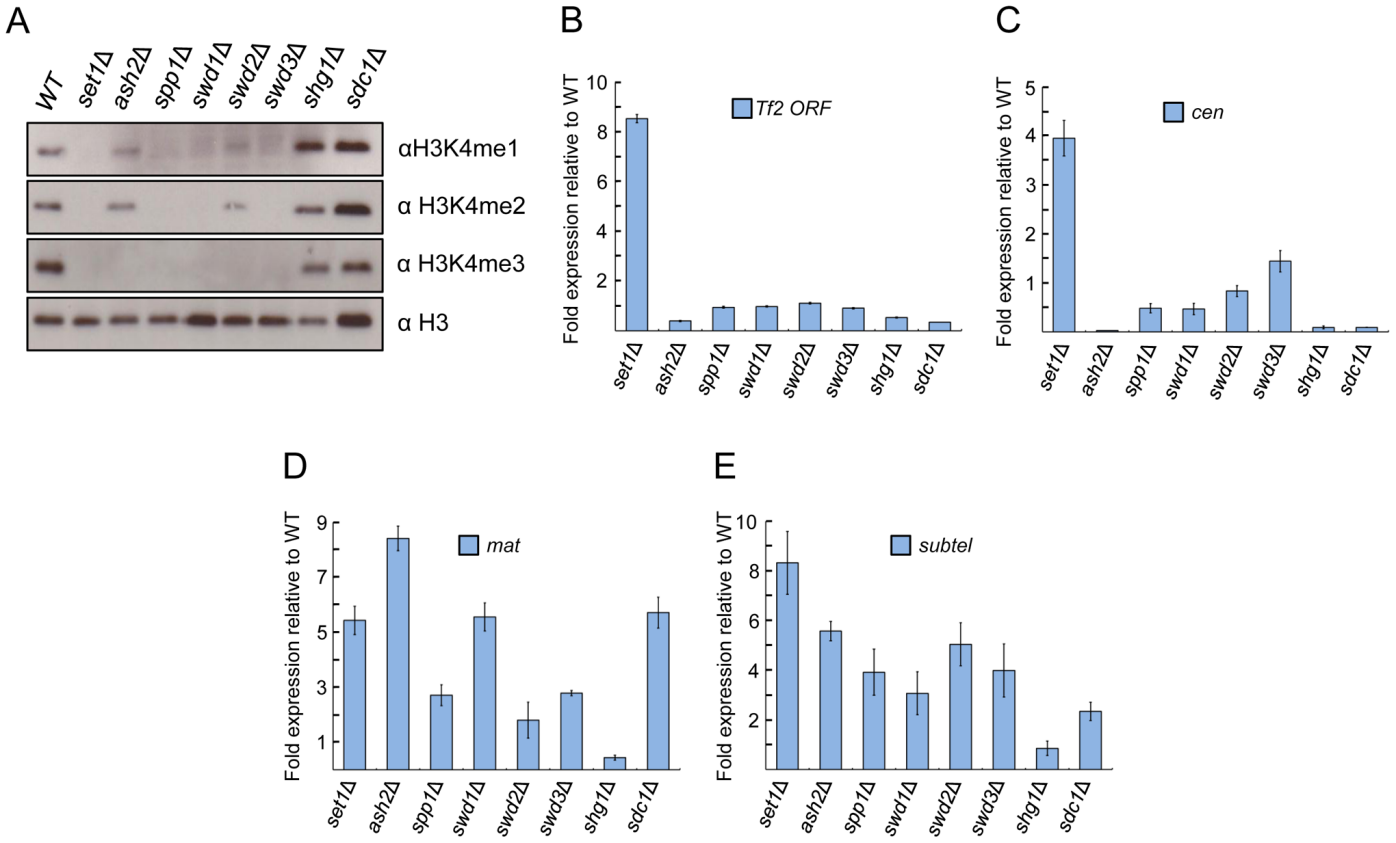
Set1 represses *Tf2s* and heterochromatic loci dependent and independent of Set1C subunits. (A) Set1C components contribute differently to H3K4me. H3K4me1, H3K4me2, and H3K4me3 were analyzed from histone extracts of indicated Set1C mutant strains by western blotting. (B) Set1-mediated repression of *Tf2s* is little affected in other Set1C mutant strains. (C–E) Set1 represses heterochromatic loci dependent and independent of Set1C. qRT-PCR was performed as in Figure 1 (s.d., error bars; n = 3). Pericentromeric repeat *dg* (*cen*), silent mating type *cenH* (*mat*), subtelomeric *prl70* (*subtel*).

The contribution of individual Set1C subunits to H3K4me has been well characterized in *S. cerevisiae* [8], [9], [10], [38], [39], [40], [41]. However, aside from H3K4me2 [24], the roles of Set1C subunits in *S. pombe* in H3K4 methylation are not well explored. We therefore assessed the status of H3K4me in cells deficient for individual Set1C components. Cells deficient for *set1*, *swd1* or *swd3* exhibit a complete loss of H3K4me (Figures 3A and S3), similar to results observed in *S. cerevisiae* mutant orthologs [41]. *spp1* (*spf1*) is essential for all three forms of H3K4me in *S. pombe*. In contrast, the loss of *S. cerevisiae SPP1* only diminishes [9], [31] or abolishes H3K4me3 [8]. Loss of *swd2*, which is lethal in *S. cerevisiae* [42], abolished H3K4me3 and diminished the levels of H3K4me2 and H3K4me1 in *S. pombe*. Cells lacking *ash2* exhibited relatively intact levels of H3K4me2 and H3K4me1. Although H3K4me3 was detectable within individual cells in the *ash2* mutant (Figure S4), we were unable to detect H3K4me3 at bulk histone levels (Figure 3A). In the *sdc1* mutant, only H3K4me3 level was slightly diminished, while H3K4me2 and H3K4me1 levels were largely unaffected. These results differ from budding yeast findings, in which loss of *ASH2* or *SDC1* reduced all three states of H3K4me [9], [10] or completely abolished H3K4me2 [40]. All three forms of H3K4me appeared to be intact in cells deficient for *shg1*. Collectively, our results show that several components of *S. pombe* Set1C make different contributions to H3K4me compared to their orthologs in *S. cerevisiae*.

### Set1C subunits exhibit distinct effects on transcriptional repression of *Tf2s* and heterochromatic repeats

To gain insights into potential mechanisms underlying the repressive function of Set1, we investigated the role of individual Set1C subunits in the repression of *Tf2s* and heterochromatic repeats. We found that loss of repression at *Tf2s* and pericentromeric repeats was observed only in the *set1*Δ mutant (Figures 3B and 3C). However, at the mating-type and subtelomeric regions, cells deficient for any of the Set1C components except *shg1* displayed a noticeable derepression (Figures 3D and 3E). These findings indicate that depending on genomic context, Set1 can dispense or act together with Set1C components to repress different classes of repetitive elements.

### Set1 stability is differentially affected in the absence of Set1C subunits

**Figure 4:**
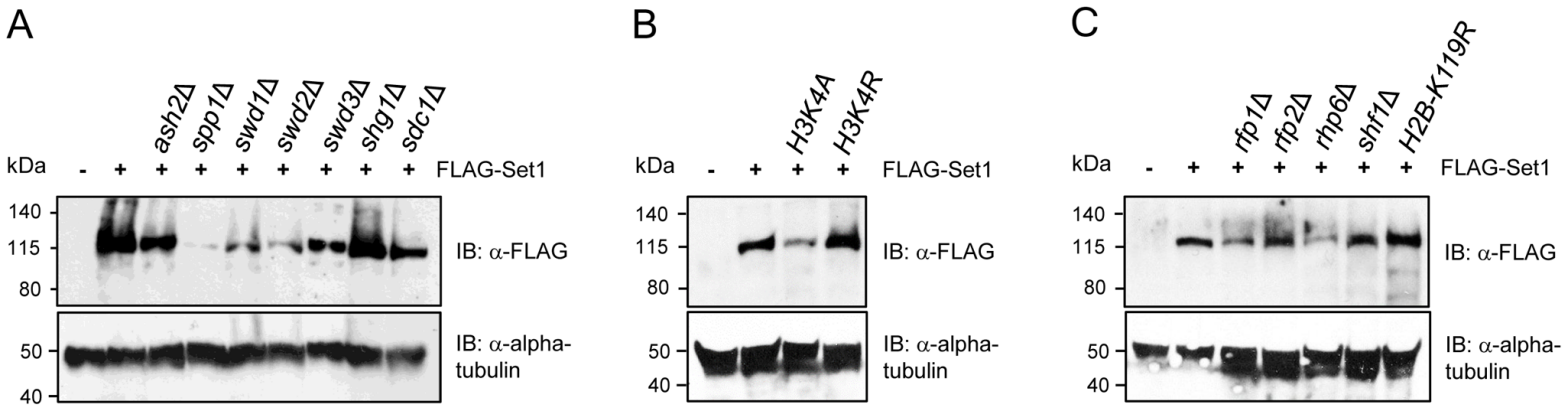
Stability of Set1 proteins is uncoupled from the status of H3K4me and H2Bub. Set1 proteins containing an N-terminal FLAG epitope were analyzed by immunoblotting (IB) in cells deficient in (A) individual components of Set1C, (B) histone H3K4 mutants, or (C) HULC/H2Bub mutants. Alpha tubulin (loading control) was detected by anti-tubulin antibody (tat-1).

In budding yeast, reduced levels of Set1 proteins have been observed in mutants deficient in *SWD1*, *SWD2*, *SWD3*, or *SPP1* [10], [11], [38], [43], [44]. We examined the levels of Set1 proteins in *S. pombe* strains lacking each of the other Set1C components. The levels of Set1 were minimally affected by the loss of *ash2*, *sdc1* and *shg1*, somewhat diminished in *swd1*Δ and *swd3*Δ, and most noticeably reduced in *swd2*Δ and *spp1*Δ mutants (Figure 4A). The loss of individual Set1C subunits on Set1 protein stability is likely to occur at the posttranslational level, as Set1 transcript levels were relatively unaffected in Set1C mutants (Figure S5).

### Set1 protein abundance is not coupled to H3K4 methylation or H2B ubiquitination

Soares *et al.* recently reported that there is feedback control linking the stability of Set1 proteins to the status of H3K4me in *S. cerevisiae* [12]. Our analysis showed that despite the complete loss of H3K4me in *set1F ^H3K4me-^* or *set1* mutants lacking either the *SET* or *pSET* domain, the levels of these Set1 mutant proteins remain comparable to that of wildtype (Figure S2). However, except for *ash2*Δ, reduced Set1 protein levels in Set1C mutants (i.e., *spp1*Δ, *swd1*Δ, *swd2*Δ, *swd3*Δ) generally correlated with a loss of H3K4me, in particular, H3K4me2/3 (see Figure 3A). To disentangle the effects of Set1C component deficiency or Set1 mutations on the stability of Set1 proteins from their contributions to Set1 activity toward H3K4me, we analyzed Set1 proteins in H3K4 mutant cells. We found that Set1 proteins are readily detectable in either H3K4A or H3K4R strains though with apparently reduced levels in H3K4A mutant (Figure 4B). Mono-ubiquitination of histone H2B (H2Bub) has been shown to contribute to H3K4 methylation [45], [46], [47], [48], [49]. In *S. pombe* H2Bub is mediated by a histone H2B-conjugating complex termed HULC, consisting of a *rad6* ortholog Rhp6, two RING finger proteins Rfp1 and Rfp2 similar to budding yeast Bre1, and a serine-rich protein Shf1 [50], [51]. Deficiency of HULC components resulted in drastic reduction in H3K4me levels [50], [51]. We analyzed the loss of H2Bub or HULC subunits on the abundance of Set1 proteins. We could detect Set1 proteins in all HULC/H2Bub mutants. Similar to H3K4A, Set1 levels were reduced in *rhp6*Δ (Figure 4C). Our results suggest that in contrast to what has been observed in *S. cerevisiae*, the regulation of Set1 protein abundance in *S. pombe* is largely independent of the status of H3K4 methylation and H2B ubiquitination.

### Set1 regulates the nuclear organization of *Tf2s* distinct from its transcriptional repressor function

**Figure 5:**
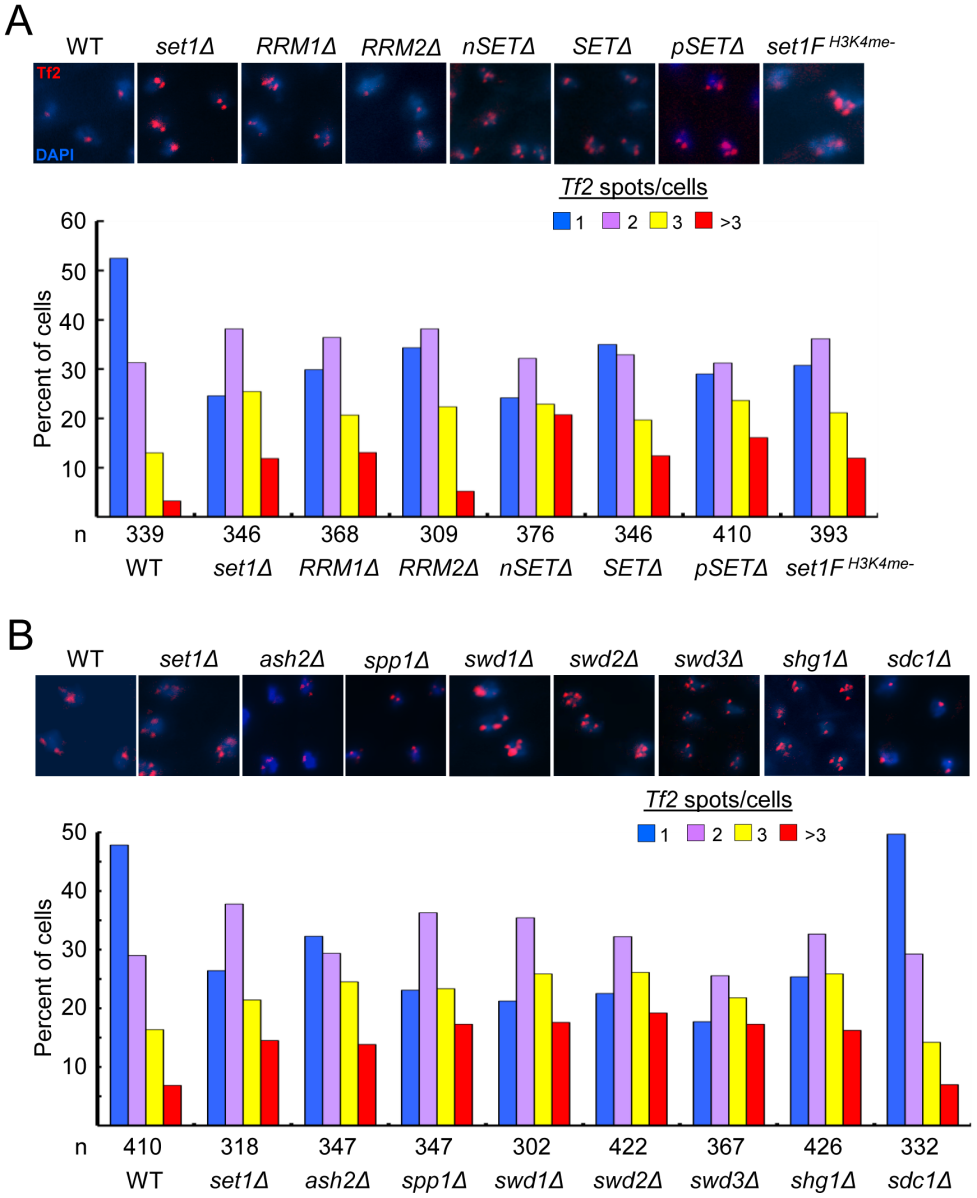
Set1C and H3K4me are required for the integrity of *Tf* bodies. (A, B) Declustering of *Tf2s* in (A) *set1* mutant with defects in H3K4me and (B) Set1C mutants. Fluorescence *in situ* hybridization (FISH) analysis was performed using a FISH probe corresponding to the ∼3.6 kb coding region of *Tf2* elements. Representative FISH images from indicated strains (top panels). Quantitative FISH analysis of observed *Tf2* foci/cell in indicated strains (bar graph; bottom panels). Number of cells analyzed per strain (*n*). Except for *sdc1*Δ, *Tf2* declustering in all mutant strains compared to WT was significant (*p*<0.005, chi-square test).

We have previously identified a novel role for Set1 in the nuclear organization of *Tf2s* into *Tf* bodies [27]. To dissect potential mechanisms underlying Set1-mediated clustering of *Tf2s*, we performed fluorescence *in situ* hybridization (FISH) analysis to monitor the status of *Tf* bodies in various *set1* mutants. In contrast to wildtype cells with intact *Tf* bodies, *set1* mutants lacking the RRM1, nSET, SET or pSET domain exhibited defects in *Tf* bodies to the same extent as *set1*Δ (Figure 5A). Because H3K4me is severely compromised in these *set1* mutants (see Figure 1B and. S1), H3K4me might be required to maintain the integrity of *Tf* bodies. Indeed, *Tf2* elements were observed to decluster in the *set1F^H3K4me-^* cells which lack H3K4me altogether. In the *set1* mutant containing the RRM2 deletion, which resulted in intermediately increased levels of *Tf2* expression (see Figure 1C), the integrity of *Tf* bodies was modestly affected. These results suggest that Set1 relies on disparate domains and possibly different catalytic activities (see discussion below) to exert control over different aspects of *Tf2* regulation.

### The Set1C complex maintains *Tf* body organization by antagonizing the H3K4 histone acetyltransferase Mst1

The dependence on H3K4 methylation to maintain the integrity of *Tf* bodies suggests a role for Set1C subunits in the organization of *Tf2s* within the nucleus. We utilized *Tf2* FISH analysis to monitor the status of *Tf* bodies in strains with null mutations for each of the Set1C components. Similar to what was observed in *set1* mutants with impaired H3K4me, Set1C mutants with gross defects in H3K4me displayed declustering of *Tf2s* compared to wildtype (Figure 5B). Only in *shg1* mutant cells did we observe a disruption of *Tf* body integrity that did not correspond with the loss of H3K4me (Figure 3A), suggesting a distinct role for Shg1 in nuclear organization.

**Figure 6:**
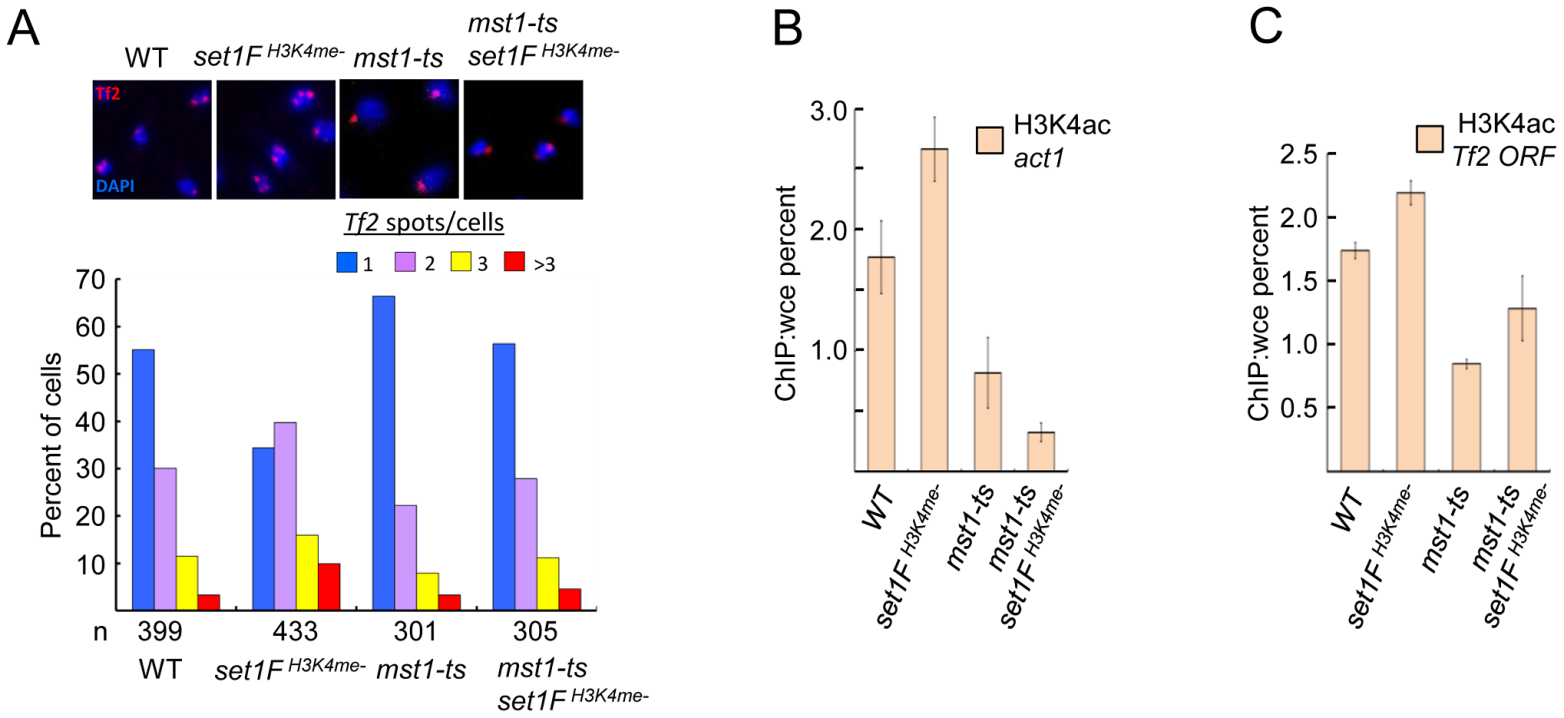
Set1C-mediated H3K4me contributes to the integrity of *Tf* bodies by antagonizing the H3K4 acetyltransferase Mst1. (A) An *mst1* mutation (*mst1-ts*) alleviates *Tf* body defects seen in an H3K4me mutant strain. Representative FISH images from indicated strains (top panels). Quantitative FISH analysis of observed *Tf2* foci/cell in indicated strains (bar graph; bottom panels). *Tf2* declustering for *set1F^H3K4me^* ^-^ strain was significant (*p*<0.005, chi-square test). H3K4ac enrichment at (B) the housekeeping gene *act1* and (C) *Tf2 ORF* in indicated mutant strains was analyzed by ChIP followed by qPCR.

The MYST family histone acetyltransferase Mst1 has been shown to acetylate lysine 4 of histone H3 (H3K4ac) [37]. Declustering of *Tf2* elements in H3K4me mutants could be due to inappropriate Mst1 activity such as heightened H3K4ac at *Tf2s*. Consistent with this idea, mutation of *mst1* in cells lacking H3K4me abrogated defects in the integrity of *Tf* bodies (Figure 6A) and diminished the elevated H3K4ac levels at *Tf2s* and the housekeeping gene *act1* observed in the absence of H3K4me (Figure 6B and 6C). Together, these results reveal that Set1 controls *Tf2* repression independent of the Set1C complex, but relies on Set1C-mediated H3K4me to maintain *Tf* body integrity by antagonizing the H3K4 acetyltransferase activity of Mst1.

## Discussion

### The contributions of Set1 domains and Set1C subunits to H3K4 methylation in *S. pombe*

Unlike other chromatin-modifying enzymes such as the Clr4/Suv39h H3K9 methyltransferase not universally present in eukaryotes, the protein architecture of Set1 and its associated complex subunits are remarkably conserved across known eukaryotic lineages [7]. The multi-domain structure of Set1 suggests its ability to interact with multiple proteins and integrate opposing inputs. Works from budding yeast have yielded many insights into the roles of various Set1 domains in H3K4me and domain interactions with various components of the Set1C/COMPASS complex [10], [11], [28], [29], [30]. Our analysis of Set1C in *S. pombe* reveals additional insights. We found that the nSET, SET, and pSET domains are essential for all three states of H3K4me, similar to results previously noted in budding yeast [12], [28]. In the RRM1 mutant, we observed defects in H3K4me2 as previously reported [22], in addition to the complete loss of H3K4me3 and slightly reduced H3K4me1, though the degree to which these defects are due to reduced Set1 abundance in this mutant is unclear. Reduced levels of Set1 proteins in RRM1 and nSET mutants could also account for the relatively lower enrichment of these mutant proteins at euchromatic targets observed in ChIP experiments. In contrast, the loss of RRM2 did not appear to affect the protein levels of Set1 or hamper the ability of Set1 to methylate all three states of H3K4me. In fact, we detected slight increases in overall H3K4me levels in the RRM2 mutant (Figure 1B), suggesting it might have an inhibitory role against H3K4me, similar to the roles of the central autoinhibitory region noted in budding yeast Set1 [28].

The contributions of several *S. pombe* Set1C subunits to H3K4me diverge from those in *S. cerevisiae*. Among these are Ash2, Sdc1, Swd2, and Spp1 (Spf1). *S. pombe spp1* is essential not only for H3K4me2 [24], but H3K4me3 and H3K4me1. This pattern is somewhat similar to that seen in *S. cerevisiae spp1*Δ mutant expressing a Set1 C-terminal fragment containing only the nSET, SET, and pSET domains [11], [30]. These data point to a more critical role for Spp1 in conferring the H3K4me activity within the Set1C complex in *S. pombe* compared to *S. cerevisiae*, perhaps by more effectively countering the inhibitory effect of the RRM2 domain on H3K4me and/or by influencing the conformation of the nSET domain within the Set1C complex to effect H3K4me [11], [30]. However, the substantially reduced amount of Set1 protein levels in *S. pombe spp1* null cells could also contribute to the complete loss of all three forms of H3K4me. Differences in the role of the Swd2 subunit in previous reports in budding yeast and our study were also noted. *S. cerevisiae* mutant cells carrying temperature-sensitive alleles of *SWD2* exhibit severe reductions in H3K4me2/3 [43], [52], and an *S. cerevisiae swd2*Δ mutant overexpressing a C-terminal fragment of Sen1 that suppresses the lethality of *swd2*Δ displays similar defects in H3K4me2/3 but no significant change to H3K4me1 [44]. In contrast, we found reduction for all three states of H3K4me in *S. pombe swd2*Δ cells, with H3K4me3 most affected, followed by H3K4me2 and H3K4me1. Similar H3K4me2/3 defects in both yeast species deficient in *swd2* could be due to the requirement of *swd2* to maintain sufficient levels of Set1 protein abundance [43], [44]. However, H3K4me1 defects seen in *S. pombe swd2*Δ might reflect a more dedicated role for Swd2 in Set1C function compared to its *S. cerevisiae* counterpart, which is also a subunit of the essential transcription termination factor APT [53], and proposed to be needed to overcome antagonism by Set1C [54]. Despite discrepancies in findings from several groups [9], [10], [11], [40], [44], *S. cerevisiae ash2*Δ and *sdc1*Δ mutants exhibit similar H3K4me defects, likely reflecting their function as a heterodimer within the Set1C complex [10], [11]. Our study suggests a more extensive role for *S. pombe* Ash2 than Sdc1 in maintaining various states of H3K4me, in particular H3K4me3 and H3K4me2. In addition, loss of either *ash2* or *sdc1* did not produce H3K4me defects as severe as those seen in the equivalent budding yeast mutants. These differences might reflect divergence in the functions of Ash2 and Sdc1 in *S. pombe*, likely due to their associations with the histone H3K4 demethylase Lid2 complex, which is not present in *S. cerevisiae* [24], [55].

### Set1 stability and its relation to H3K4me

Set1 has been documented to be a highly unstable protein in budding yeast [44]. This instability of Set1 has been shown to be coupled to the levels of H3K4me [12], complicating efforts to untangle the specific roles of various Set1 domains and Set1C subunits to H3K4me from their direct contributions to the stability of Set1. Our study indicates that Set1 is inherently more stable in *S. pombe*, and is readily detectable in whole cell extracts from wildtype and mutant strains deficient for either the individual Set1 domains or Set1C complex components. It is worth noting that the RRM1 and nSET domains and certain Set1C subunits (Swd1, Swd2, Swd3, Spp1) appear to contribute to Set1 stability. However, the regulation of Set1 protein levels in *S. pombe* is largely independent of H3K4me abundance and H2Bub levels, further highlighting the considerable divergence in the regulation of Set1 in *S. pombe* versus that of *S. cerevisiae*.

### Set1-mediated repression of *Tf2s* and heterochromatic loci

**Figure 7:**
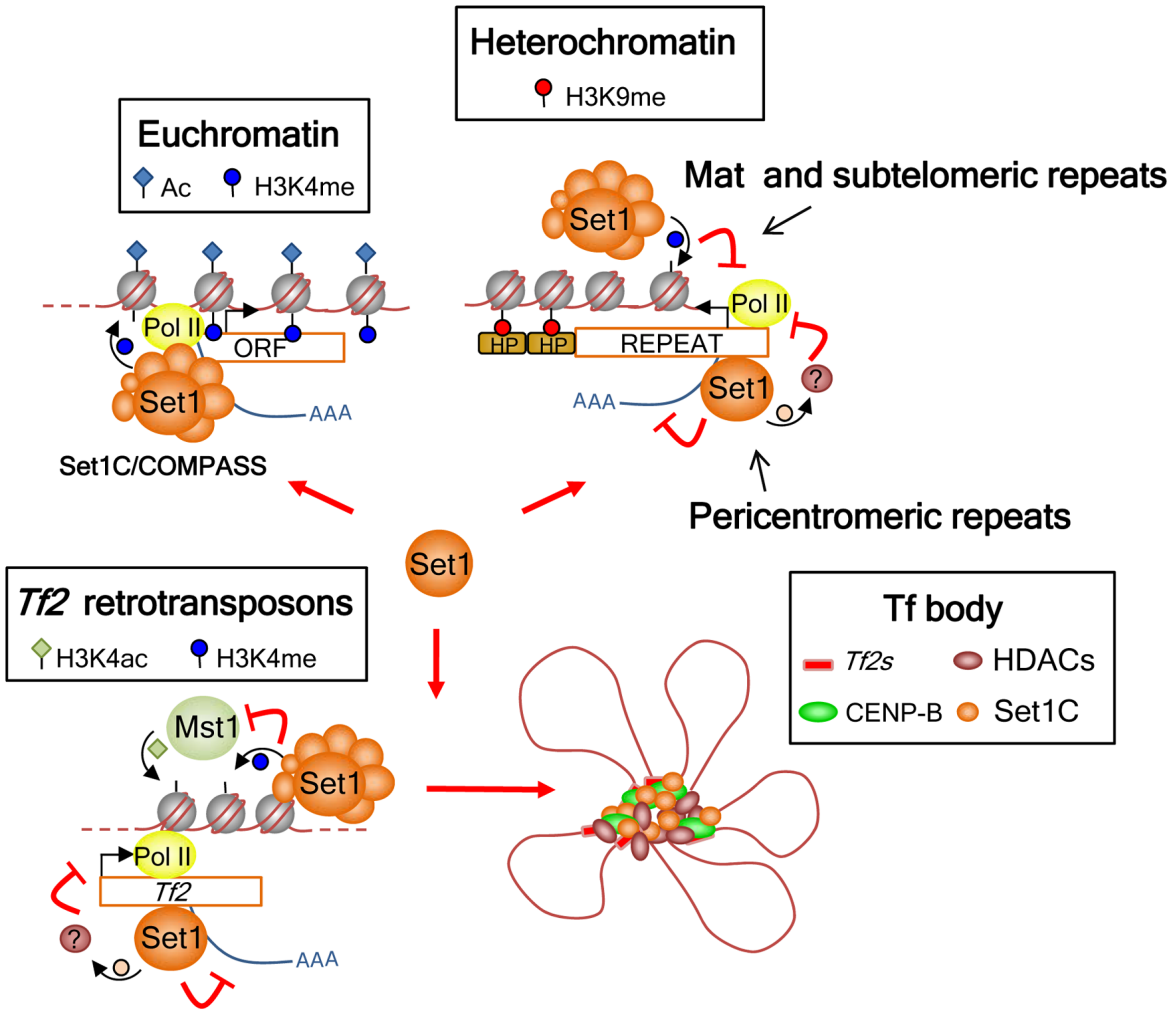
Model for the roles of Set1C in genome control in *S. pombe*. Set1 exerts its multifaceted genome control at euchromatin and heterochromatin dependent and independent of H3K4 methylation and the Set1C complex. At euchromatin, Set1 operates as part of the Set1C/COMPASS complex that is responsible for H3K4me distribution at active RNA polymerase II genes. At interspersed *Tf2s* and heterochromatic loci, Set1 has a repressive role mediated through two distinct pathways: H3K4me/Set1C-dependent repression at the silent *mat* locus and subtelomeres, and H3K4me/Set1C-independent repression at *Tf2s* and pericentromeric heterochromatin. Our findings also anticipate the presence of novel non-histone H3K4 substrates involved in the repression of *Tf2s* and pericentromeric repeats that could be partly mediated via Set1 association with Pol II nascent transcripts. In addition, Set1C and H3K4me have a distinct genome organization role at *Tf2s* by antagonizing the activity of the histone H3K4 acetyltransferase Mst1 to maintain the integrity of *Tf* bodies.

The role of Set1 as a transcriptional repressor has been widely documented in budding yeast [17], [18], [20], [32], [46], [56], [57], [58]. However, these studies ascribed Set1 repressor function solely to H3K4me2 and/or H3K4me3 [19], [20], [21], [58]. We have previously shown that Set1 mediates repression of *Tf2* retrotransposons independent of H3K4me [27]. Our current study reveals an unanticipated complexity in the repressive function of Set1, in that the requirement of H3K4me in transcriptional silencing depends upon the genomic context (Figure 7). The complete loss of H3K4me does not appear to hamper the ability of Set1 to localize to and maintain repression at *Tf2s* and pericentromeric repeats, while at the *mat* locus and subtelomeric repeats Set1-mediated H3K4me contributes to repression. These findings were consistent with analyses using Set1C subunit deletion mutants. Loci that depend on H3K4me-mediated repression (*mat* and subtelomeric repeats) also require Set1C components needed for maintaining proper H3K4me (all Set1C subunits except Shg1). Repression of *Tf2s* and pericentromeric repeats, on the other hand, is maintained in all Set1C mutants (except *set1*Δ).

Our study identifies a novel mode of Set1 function that does not depend on H3K4me and an intact Set1C complex. The requirement of the SET domain but not H3K4me activity suggests that Set1 mediates repression of *Tf2s* and pericentromeric repeats via methylation of a novel substrate(s). In budding yeast, Set1 has been shown to methylate Dam1, a component of the kinetochore DASH complex [59]. Although methylation of Dam1 is independent of H3K4me, its methylation requires other Set1C subunits [40]. *S. pombe* encodes a *dam1* ortholog, though it appears not to contain conserved Set1 methylation sites [60]. Considering that Set1 represses *Tf2s* and pericentromeric repeats independent of H3K4me and other Set1C subunits, it is possible that repression of these repeats might involve Set1 binding to RNA via its RRM2 domain and methylation of targets associated with either transcription and/or RNA processing.

Heterochromatic repeats and *Tf2s* in certain genetic backgrounds are targeted for RNAi-mediated heterochromatic and exosome-mediated silencing [26], [61], [62]. It has been shown that histone deacetylases (HDACs) cooperate with RNAi to assemble heterochromatin at pericentromeres [63]. Even though loss of *set1* does not appear to affect the levels of H3K9 methylation and siRNAs at pericentromeric heterochromatin (Figure S6A) [37], there were noticeable increased levels of H3K9 acetylation at that region (Figure S6B). Several HDAC mutants (i.e., *sir2*, *clr3*) are known to retain robust levels of siRNAs and H3K9me and yet exhibit increased levels of certain histone acetylation marks at pericentromeres [63], [64], [65]. Thus, it is likely that in the absence of *set1*, HDACs, RNAi and exosome act in redundant pathways to help maintain heterochromatin.

### The roles of Set1C in genome organization

We previously reported that Set1 has a novel role in genome organization by clustering interspersed *Tf2* elements into *Tf* bodies [27]. Even though declustering of *Tf2s* was not observed in H3K4 mutant strains (H3K4A, H3K4R) [27], a role for H3K4me in *Tf2* clustering could not be excluded due to the loss of both H3K4 acetylation and methylation in those H3K4 mutants. Our current study supports an active role for the Set1C complex in maintaining the integrity of *Tf* bodies by antagonizing the function of the H3K4 acetyltransferase Mst1. Set1 has been shown to limit the abundance of H3K4ac at gene promoters in *S. cerevisiae* [66]. Thus, H3K4me catalyzed by Set1C could compete with Mst1-mediated H3K4ac at *Tf2s* to maintain the integrity of *Tf* bodies. However, as loss of H3K4me also results in increased H3K4ac at the house keeping gene *act1*, *Tf2* declustering in *set1* mutants could reflect global changes in genome organization due to heightened levels of H3K4ac across multiple loci. HDACs recruited by CENP-B proteins to *Tf2s* have also been shown to contribute to *Tf2* clustering [27], [67]. Cells may therefore exploit dynamic competition between Set1C, HATs, and HDACs to regulate the various states of H3K4, which could in turn facilitate rapid genome reorganization in response to acute environmental changes [68].

## Materials and Methods

### Strain construction

Null mutant and C-terminal FLAG (3×) strains were constructed using a Kanamycin cassette [69]. Double mutants were generated by standard genetic cross methods [70]. Full-length and domain mutants of *set1* containing an N-terminal FLAG (3×) epitope were generated by a two-step site-directed mutagenesis (SDM). First, the *set1* gene was replaced with a *ura5 lys7* cassette [71]. Second, an SDM PCR fragment containing either full-length or domain deleted FLAG-Set1 was transformed into the above *set1* null strain (*set1*Δ::*ura5 lys7 ura5-14 lys7-2*), and transformants were scored by growth on the uracil counter selective agent 5-Fluoroorotic acid (5-FOA) and sensitivity to lysine minus media [71]. Proper insertions were confirmed by PCR and DNA sequencing. Liquid cultures were grown at 30°C in standard rich media supplemented with 225 mg/L adenine (YEA).

### Quantitative Reverse Transcription Real-time PCR (qRT-PCR)

RNA was isolated by a hot acid phenol method [72] and converted to cDNA with Superscript III reverse transcriptase and anchored oligo-dT primer (Life Technologies). cDNA was subjected to qPCR analysis using DyNAzyme™ II PCR Master Mix (Finnzymes) with SYBR green on the Applied Biosystems 7500 Fast Real-Time PCR System. Fold expression changes of mutant versus wildtype cells relative to *act1* gene were determined using the 2^−ΔΔCt^ method in Microsoft Excel.

### Histone extraction and detection

Cells from 50 ml culture (OD∼0.5) were washed in 10 ml NIB buffer (15 mM PIPES pH 6.8, 0.25M sucrose, 60 mM KCl, 15 mM NaCl, 5 mM MgCl_2_, 1 mM CaCl_2_, 0.8% Triton X-100, 10 ng/µl TSA, 1 mM PMSF, Roche protease inhibitor mini tablet), lysed with acid-washed glass beads in a bead beater, and centrifuged at 11,000× *g* for 10 min [22]. Cell extract pellets were resuspended in 0.4M H_2_SO_4_, incubated on ice for 1 h with occasional mixing, and the supernatant was collected following centrifugation at 8,000× *g* for 5 min. Histone extract was concentrated by trichloroacetic acid (TCA) precipitation, washed in acetone and resuspended in 100 µl LDS buffer (Life Technologies) and quantitated using the BCA method (Pierce). 5 µg of histone extracts were resolved on 14–22% Tricine SDS PAGE and transferred to a nitrocellulose membrane using the iBlot system (Life Technologies). Histone H3 and modified residues were detected with antibodies against H3 (Abcam, ab1791), H3K4me1 (Abcam, ab8895), H3K4me2 (Fisher, 07030MI), or H3K4me3 (Fisher, 07-473MI).

### Protein extractions and western blotting

*S. pombe* cells (OD 1–2) were lysed in HCS buffer (150 mM HEPES pH 7.2, 250 mM NaCl, 0.1% NP-40, 1 mM EDTA, 1 mM dithiothreitol, 1 mM PMSF) and protein inhibitor tablet (Roche) by acid-washed beads in a bead beater (three times 30 s with 2 min interval on ice). 50 µg of protein extracts were run on a PAGE gel (Express Plus 4–20% Bis-tris (MOPS), Genescript) and subjected to overnight western blot transfer at 4°C. Set1 was detected using anti-FLAG antibody (Genescript, A00187).

### Chromatin Immunoprecipitation (ChIP)

ChIP assays were performed as previously described [27]. qPCR was performed using Phire Hot Start II DNA Polymerase (Thermo Scientific) supplemented with SYBR green (Life Technologies) on the Applied Biosystems 7500 Fast Real-Time PCR System. Enrichment of ChIP *vs.* input DNA was determined using the 2^−ΔΔCt^ method in Microsoft Excel.

### Immunofluorescence (IF) and Fluorescence *In Situ* Hybridization (FISH)

IF and FISH assays were performed as previously described [27], [68]. Briefly, *S. pombe* cells were grown in 10 ml YEA media until OD_595_ ∼0.5–1. 10 ml of 2.4 M sorbitol YEA solution was added to culture, and cells were immediately cross-linked with 2.9 ml of freshly made 30% paraformaldehyde/YEA solution for 30 min in a 18°C water bath shaker. Cross-linked reaction was quenched with 1.2 ml of 2.5 M glycine. Cells were transferred to a microcentrifuge tube, subjected to cell wall digestion in 0.5 mg/ml zymolyase solution (Associated of Cape Cod, 100T) at 37°C for 30 min, blocked with PEMBAL (100 mM PIPES pH 6.9, 1 mM EGTA, 1 mM MgSO4, 1% BSA, 0.1 M L-lysine) for 1 hr and subjected to either IF or FISH analysis. For IF analysis, cells were incubated overnight at room temperature with antibodies against either H3K4me1 (Abcam, ab8895), H3K4me2 (Fisher, 07030MI), or H3K4me3 (Fisher, 07-473MI). Cells were then incubated with anti-mouse Alexa Fluor 488 (Invitrogen) followed by DAPI staining to visualize H3K4me signal in the nucleus. For FISH analysis, PEMBAL-treated cells were treated with RNase A (0.1 mg/ml) at 37°C for 3 h. Hybridization was carried out at 40°C for 12–14 h with 100–150 ng of Tf2-orf probes in 100 µl hybridization buffer (50% formamide, 2× SSC, 5× Denhart's solution, 10% dextran sulfate). Cells were washed three times in 100 ml 2× SSC for 30 min each. Images were obtained using a Zeiss Axioplan 2 microscope. The chi-square test of homogeneity was used to determine whether declustering of *Tf2* elements seen in mutant cells relative to wildtype was significant.

## Supporting Information

Figure S1 H3K4 methylation in *set1* mutants. Mono (H3K4me1), di (H3K4me2), and tri (H3K4me3) methylation in indicated *set1* mutant strains was analyzed by Immunofluorescence (IF). Full-length or domain deletion mutants of *set1* contain an N-terminal FLAG (3×) epitope. *set1F^H3K4me-^* corresponds to a H3K4me null mutant due to the presence of a FLAG (3×) epitope at the C-terminus of Set1. Cell nuclei were visualized by DAPI staining.
(PDF)

Figure S2 Various domains of Set1 have varied contributions to protein stability. Schematic of *S. pombe* Set1 protein showing its various domains (top panel). Protein levels of Set1 in various domain deletion mutants (bottom panel). Except for *set1F^H3K4me-^*, Set1 proteins from strains expressing an N-terminal FLAG (3×) epitope attached to either full-length or domain deletion of *set1* were detected by immunoblotting (IB) with an anti-FLAG antibody. Alpha tubulin (loading control) was detected by anti-tubulin antibody (tat-1).
(PDF)

Figure S3 Repression of heterochromatic loci is largely maintained in H3K4 mutants (H3K4A, H3K4R). Expression at (A) pericentromeres, (B) silent mating type locus, and (C) subtelomeres was analyzed using qRT-PCR in indicated mutant strains. Fold changes relative to wildtype were normalized to *act1* expression. (s.d., error bars; *n* = 3). Pericentromeric repeat *dg* (*cen*), silent mating type *cenH* (*mat*), subtelomeric *prl70* (*subtel*).
(PDF)

Figure S4 H3K4 methylation in Set1C mutants. Status of mono (H3K4me1), di (H3K4me2), and tri (H3K4me3) methylation in strains null for indicated Set1C subunits was analyzed by Immunofluorescence (IF). Cell nuclei were visualized by DAPI staining.
(PDF)

Figure S5 Set1 transcript levels are not noticeably altered in strains deficient for Set1C components. RNA extracted from indicated Set1C mutant strains was converted into cDNA and used in a PCR reaction to assess RNA levels of *set1* and the actin gene *act1* (control) in corresponding Set1C mutants.
(PDF)

Figure S6 Loss of *set1* results in increased H3K9 acetylation at pericentromeric repeats. (A) Enrichment of H3K9 methylation (H3K9me2) (Abcam, ab1220) and (B) H3K9 acetylation (H3K9ac) (Millipore, 07-352) was determined by chromatin immunoprecipitation (ChIP) followed by quantitative PCR (qPCR) using primers corresponding to the pericentromeric repeat *dg* region. ChIP fold enrichment of H3K9me2 and H3K9ac at the *dg* repeat was determined relative to the corresponding enrichment at (A) the *act1* promoter and (B) the 3′ region of *act1*, respectively (s.d., error bars; n = 3 triplicates).
(PDF)
