# Supplementary figures and images for: Multifaceted Genome Control by Set1 Dependent and Independent of H3K4 Methylation and the Set1C/COMPASS Complex
Source: PLoS Genet. 2014 Oct 30;10(10):e1004740. doi: 10.1371/journal.pgen.1004740 (PMC4214589; doi:10.1371/journal.pgen.1004740)

WT

*FLAG-set1*

*RRM1Δ*

*RRM2Δ*

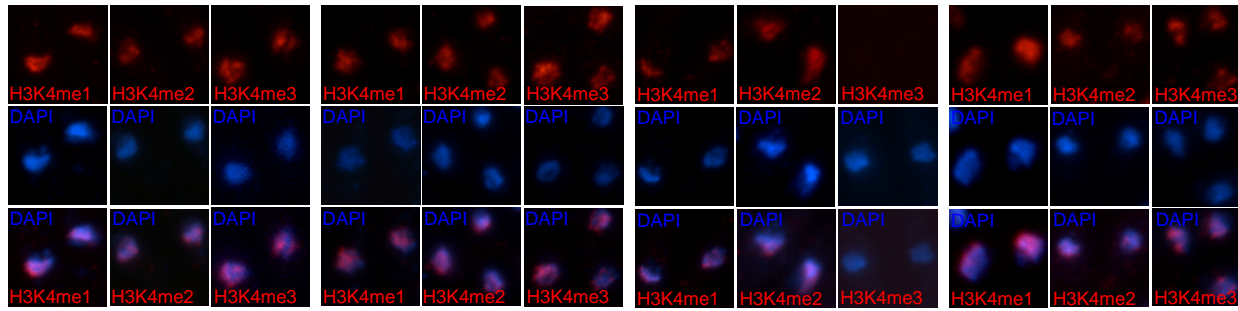

*nSETΔ*

*SETΔ*

*pSETΔ*

*set1F* <sup>H3K4me-</sup>

*set1Δ*

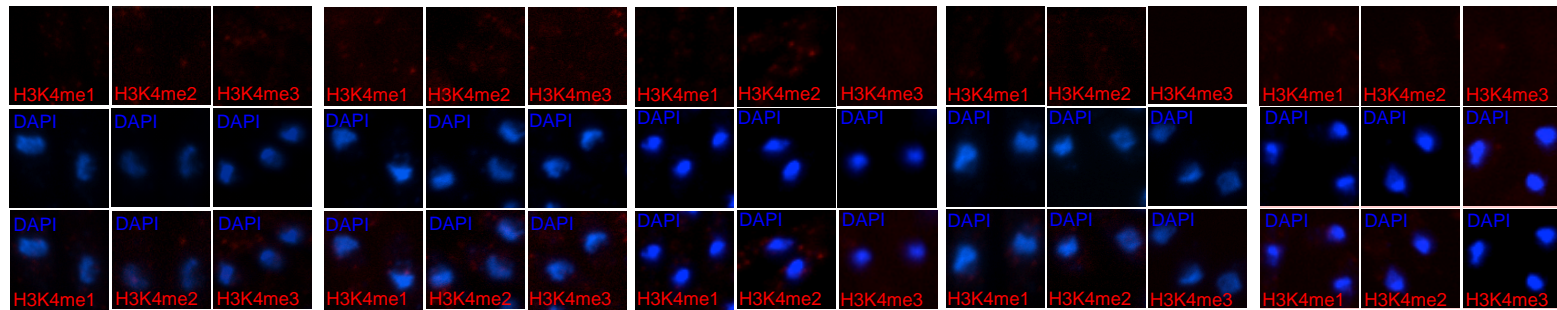

Supplement: Figure S1 — H3K4 methylation in set1 mutants. Mono (H3K4me1), di (H3K4me2), and tri (H3K4me3) methylation in indicated set1 mutant strains was analyzed by Immunofluorescence (IF). Full-length or domain deletion mutants of set1 contain an N-terminal FLAG (3×) epitope. set1FH3K4me- corresponds to a H3K4me null mutant due to the presence of a FLAG (3×) epitope at the C-terminus of Set1. Cell nuclei were visualized by DAPI staining. (PDF) [file pgen.1004740.s001.pdf]

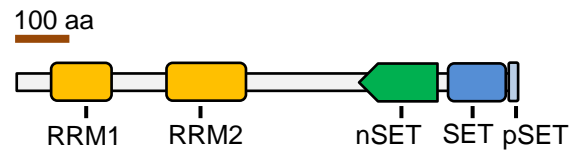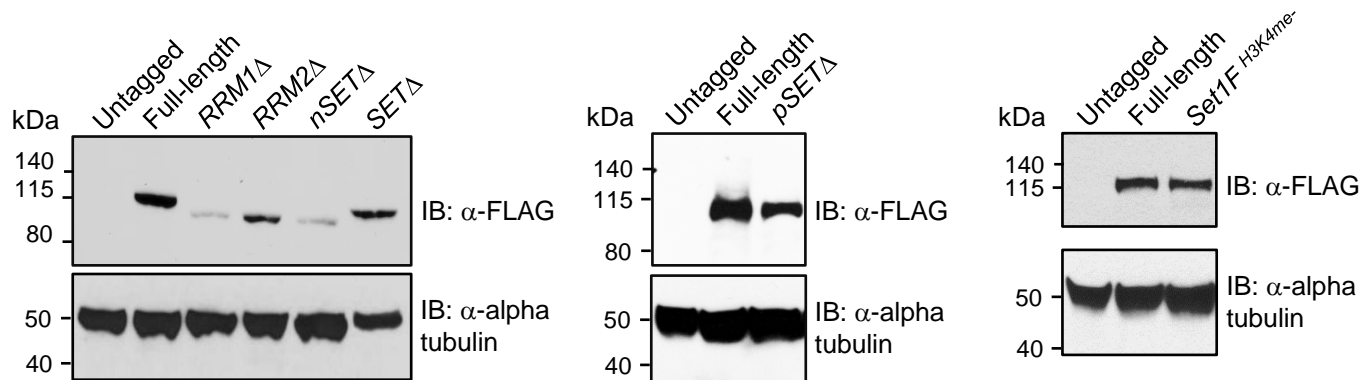

Supplement: Figure S2 — Various domains of Set1 have varied contributions to protein stability. Schematic of S. pombe Set1 protein showing its various domains (top panel). Protein levels of Set1 in various domain deletion mutants (bottom panel). Except for set1FH3K4me-, Set1 proteins from strains expressing an N-terminal FLAG (3×) epitope attached to either full-length or domain deletion of set1 were detected by immunoblotting (IB) with an anti-FLAG antibody. Alpha tubulin (loading control) was detected by anti-tubulin antibody (tat-1). (PDF) [file pgen.1004740.s002.pdf]

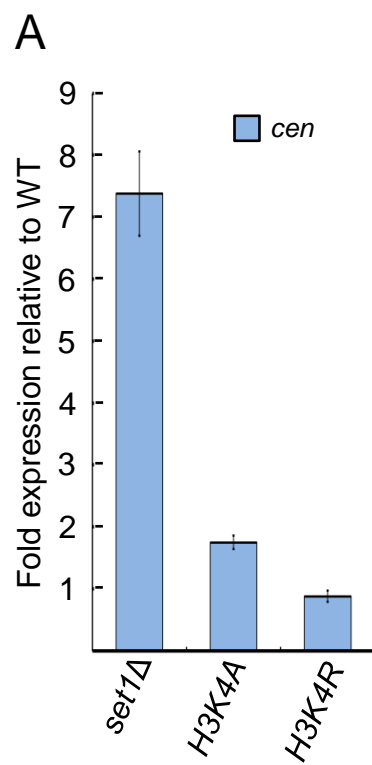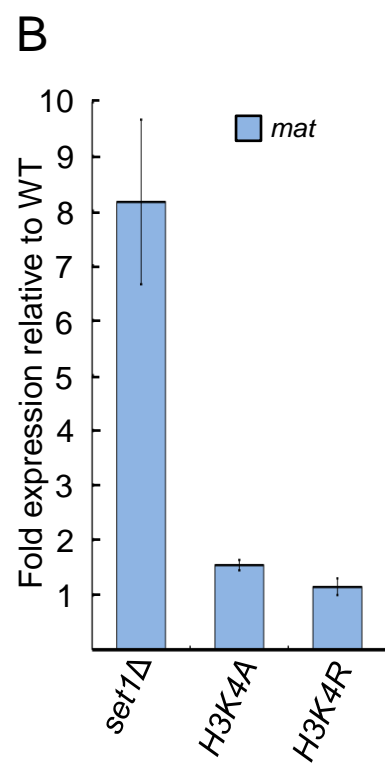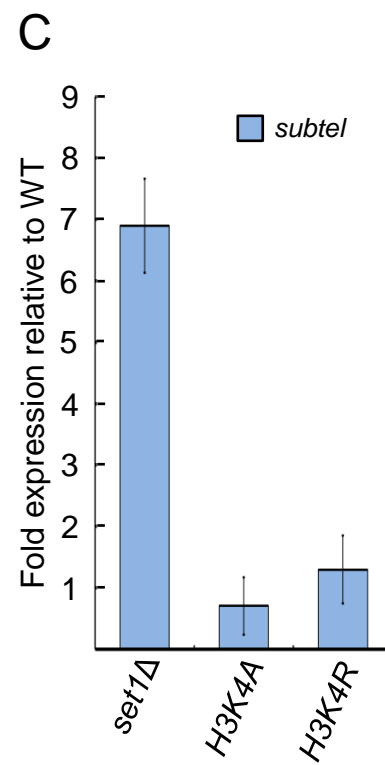

Supplement: Figure S3 — Repression of heterochromatic loci is largely maintained in H3K4 mutants (H3K4A, H3K4R). Expression at (A) pericentromeres, (B) silent mating type locus, and (C) subtelomeres was analyzed using qRT-PCR in indicated mutant strains. Fold changes relative to wildtype were normalized to act1 expression. (s.d., error bars; n = 3). Pericentromeric repeat dg (cen), silent mating type cenH (mat), subtelomeric prl70 (subtel). (PDF) [file pgen.1004740.s003.pdf]

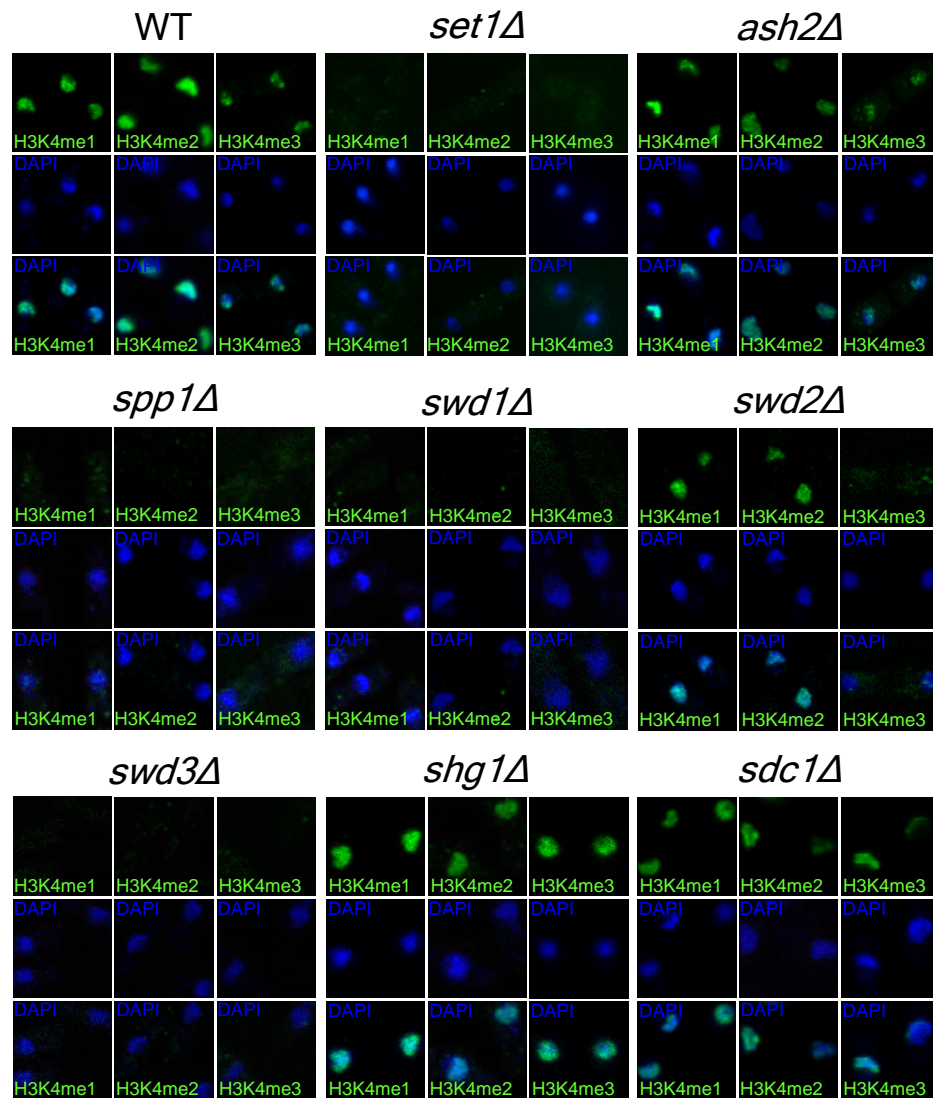

Supplement: Figure S4 — H3K4 methylation in Set1C mutants. Status of mono (H3K4me1), di (H3K4me2), and tri (H3K4me3) methylation in strains null for indicated Set1C subunits was analyzed by Immunofluorescence (IF). Cell nuclei were visualized by DAPI staining. (PDF) [file pgen.1004740.s004.pdf]

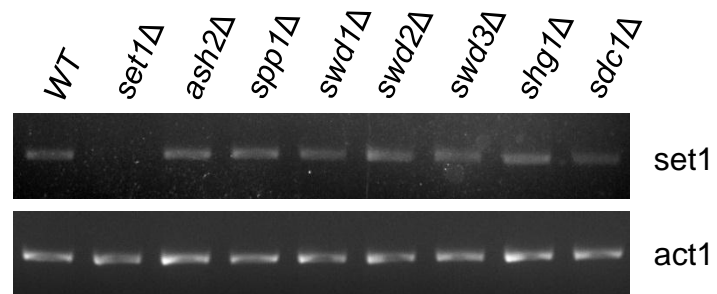

Supplement: Figure S5 — Set1 transcript levels are not noticeably altered in strains deficient for Set1C components. RNA extracted from indicated Set1C mutant strains was converted into cDNA and used in a PCR reaction to assess RNA levels of set1 and the actin gene act1 (control) in corresponding Set1C mutants. (PDF) [file pgen.1004740.s005.pdf]

A

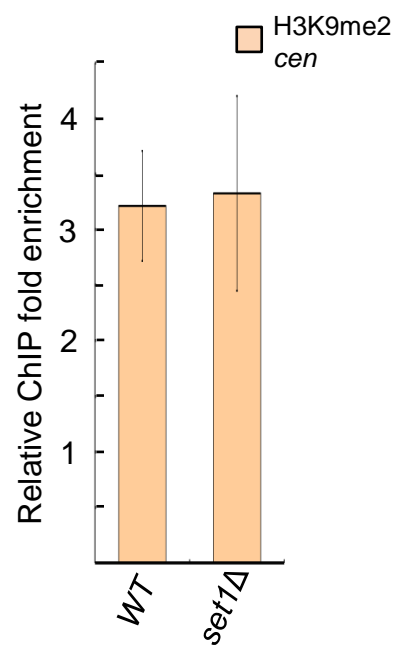

B

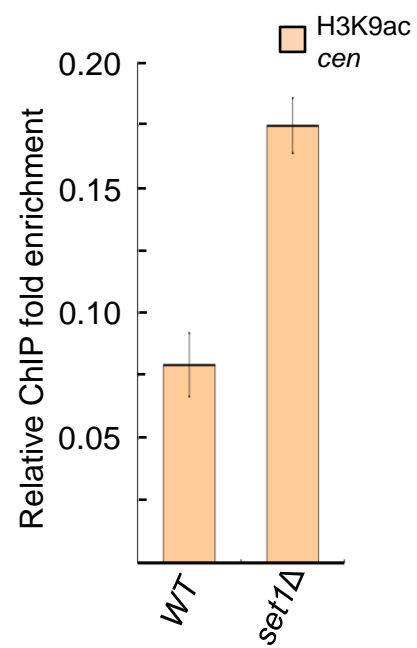

Supplement: Figure S6 — Loss of set1 results in increased H3K9 acetylation at pericentromeric repeats. (A) Enrichment of H3K9 methylation (H3K9me2) (Abcam, ab1220) and (B) H3K9 acetylation (H3K9ac) (Millipore, 07-352) was determined by chromatin immunoprecipitation (ChIP) followed by quantitative PCR (qPCR) using primers corresponding to the pericentromeric repeat dg region. ChIP fold enrichment of H3K9me2 and H3K9ac at the dg repeat was determined relative to the corresponding enrichment at (A) the act1 promoter and (B) the 3′ region of act1, respectively (s.d., error bars; n = 3 triplicates). (PDF) [file pgen.1004740.s006.pdf]
